# Establishment and performance assessment of preparation technology of internal quality control products for blood transfusion compatibility testing

**DOI:** 10.3892/etm.2013.994

**Published:** 2013-03-07

**Authors:** YANG YU, CHUNYA MA, QIAN FENG, XIN CHEN, XIAOZHEN GUAN, XIAOJUAN ZHANG, LINFENG CHEN, ZILIN LIN, JICHUN PAN, TING ZHANG, QUN LUO, DEQING WANG

**Affiliations:** Department of Blood Transfusion of PLA General Hospital, Clinical Blood Transfusion Centre of PLA, Beijing 100853, P.R. China

**Keywords:** transfusion, compatibility testing, internal quality control, red cell

## Abstract

The aim of this study was to establish and to optimize the preparation technology of whole blood internal quality control (IQC) products for blood transfusion compatibility testing. Several B-type RhD-negative blood samples collected from healthy donors were mixed. Two groups of whole blood IQC products, namely, the preservative solution group (PS group) and the saline group, were prepared. The agglutination intensity of IQC sample red cells and anti-B antibody, IgM anti-A antibody and reverse-typing A cell, IgG anti-D and O-type RhD-positive red cells, as well as free hemoglobin concentration in the supernatant of the two groups were detected. The erythrocytes in both groups were damaged to a certain extent during storage, but no evident (above moderate) hemolysis was observed in the stored sample within 42 days. The red cells remained structurally complete and the reaction activity of IgG anti-D reagent remained generally unchanged (P>0.05). Although the reaction activity oscillation of IgM anti-A reagent was observed, the agglutination intensity varied within an acceptable range of 1+. No difference was observed between the preparation methods of the samples, i.e., between the erythrocyte washed with saline and the one washed with red cell preservative solution (P>0.05). The long shelf life, low variance between tubes and stable antigen-antibody reaction activity of the whole blood IQC products prepared using the proposed method can meet the requirements of blood transfusion compatibility testing.

## Introduction

In 1950, Levey and Jennings introduced the statistical control theory of industrial product manufacturing into the clinical laboratory and established the initial clinical examination analysis quality control (QC) (1). This advancement raised the curtain for medical laboratory internal QC (IQC). Medical laboratory IQC is an indispensable part of a complete medical laboratory QC system. According to the IQC managerial system, the corresponding standards and the operation procedures, laboratory staff select suitable examination methods and procedures to evaluate consecutively the stability of the examination work performed in their laboratory. This procedure is performed to supervise and to control the precision of the examination work and to increase consistency within the same batch and between different batches. Moreover, this procedure will also determine whether the examination result is sufficiently reliable for issuance in a report, thus enabling a real-time evaluation of laboratory examination work (1–5).

Several countries have raised the requirements related to IQC of blood transfusion compatibility testing (6–9). Blood transfusion compatibility testing includes ABO classification, RhD blood grouping, irregular antibody screening and cross matching. These examinations are gradually expected to become the industry standard. Whole blood samples are used for such examinations and most IQC products are whole blood samples. Such samples are, however, more difficult to preserve and are less stable than serum or plasma IQC products that are required for traditional examinations. Given the short shelf life of commercial whole blood IQC products, the requirement of blood transfusion compatibility testing cannot be met. In this regard, blood transfusion compatibility testing laboratories have an advantage because of their access to sample resources. After blood components are issued, a few samples from these donors may still be used for the production of IQC products in the laboratory. Based on the early research on the conditions for preservation of whole blood IQC products (10), improvements have been achieved in the methods for the preparation and preservation of samples for the production of stable IQC products that can be effectively preserved and used for laboratory IQC. The preparation methods and the performance evaluation results of IQC products are given in the subsequent sections.

## Materials and methods

### Sample preparation

This study was conducted in accordance with the declaration of Helsinki and with the approval of the Ethics Committee of PLA General Hospital (permit number 20060828001). Written informed consent was obtained from all participants. All blood specimens were extracted from healthy donors, whose alanine aminotransferase, hepatitis B surface antigen, hepatitis C virus antibody, human immuno-deficiency virus (HIV) antibody and syphilis antibody all met the donor health examination requirements. The whole blood specimens were collected within 10 days prior to the analysis. No hemolysis or abnormal agglutination was observed following centrifugation. Both irregular antibody screening and the direct antiglobulin test showed negative results.

### Evaluation of the titer of commercial IgG anti-D reagent

A concentration of 100 *μ*l IgG anti-D was multiplied, continuously diluted with saline and evaluated with an as-prepared 1% O-type RhD-positive erythrocyte suspension. Micro-column gel anti-globulin cards were then used to determine the concentration of dilution when the last agglutination strength of 2+ appeared.

### Preparation process of IQC samples

Several B-group RhD-negative samples from healthy donors were selected for mixing and centrifuged at 1760 × g for 5 min. The supernatant was again subjected to 1760 × g centrifugation for 5 min to remove precipitates. The fluid was used to prepare a plasma pool. The remainder of the hematocrit-mixed erythrocytes were divided into two groups. One group was washed twice with a preservative (centrifugation condition, 1760 × g; 5 min), while the other group was washed twice with saline (centrifugation condition, 1760 × g; 5 min). The two groups of erythrocytes were mixed with a preservative and the prepared plasma pool in a volume proportion of 1:2:3, respectively.

Gentamicin sulphate 0.05 mg/ml was always added to IQC products. Commercial IgG anti-D reagent was added to IQC products at the dilution rate of the initially occurring agglutination of 2+. The two groups of modified whole blood samples were placed in hard plastic tubes, with 10 products in each group. The tubes were sealed with a cap and preserved at 4°C, with 1 h exposure at room temperature daily.

### Preparation of reagent erythrocyte for reverse-typing

Five units of 0.5 ml group A, B and O, RhD-positive hematid were randomly selected. Once the groups were mixed, the erythrocytes were washed three times with saline (centrifugation conditions, 1760 × g; 5 min). ABO forward grouping was then implemented on the erythrocytes from types A, B and O, respectively, mixed with anti-A, anti-B and standard serum. When grouping was confirmed, the erythrocytes were diluted with saline to obtain a 1% reverse-grouping erythrocyte reagent. The type-O hematocrit was prepared with low ionic strength solution (LISS) to obtain a ready-to-use 1% erythrocyte suspension. All reagent erythrocytes were prepared on the examination day.

### Performance index measurement

Measurements were taken at 0, 35, 42 and 49 days after the preparation of the IQC products, including the items below.

### Erythrocyte B antigen, plasma IgM anti-A and IgG anti-D reaction activity determination

A WADiana DG-57 (Diagnostic Grifols, Barcelona, Spain) automatic blood matching system, DG Gel ABO-CDE blood type card and DG Gel Coombs card (Grifols), as well as the as-prepared 1% A1 and B reverse-grouping reagent erythrocyte and O-group RhD-negative 1% erythrocyte suspension were used for ABO forward and reverse grouping. The RhD grouping determination and irregular antibody screening of all the IQC products (used to examine the erythrocyte B antigen), plasma IgM anti-A and IgG anti-D reaction activity, as well as all agglutination intensity data, were recorded and scored statistically. The scoring criteria (11) had six grades: Grade 5, 12 scores; Grade 4, 10 scores; Grade 3, 8 scores; Grade 2, 5 scores; Grade 1, 3 scores; and Grade 0 (negative), 0 score.

### Determination of Na^+^, K^+^, lactate dehydrogenase (LDH) and lactic acid

A VITROS 5.1 FS Chemistry System and the auxiliary reagent were used in the evaluation (Johnson & Johnson, New Brunswick, NJ, USA).

### Free hemoglobin determination of the supernatant fluid of IQC products

The Trinder reaction method was adopted. A concentration of 0.05 ml plasma was added to the examining tube, HB working solution was added into the standard tube and 0.05 ml saline was added into the empty tube. A concentration of 2.5 ml chromogen buffer was added into each tube and mixed with the contents. Subsequently, 0.5 ml 0.29 mol/l H_2_O_2_ was added to the mixtures, which were kept at room temperature for 20 min. The tube samples were examined using Multiskan-Mk3 ELISA and the color comparison was read at 492 nm. The following is the obtained result: FHb (mg/l) = (A_examining_-A_empty_) / (A_standard_-A_empty_) × 100.

Hemolysis may occur in the samples during preservation. The hemolysis condition was divided into four grades according to FHb content in the supernatant fluid. The four grades are as follows: Grade 0, no hemolysis (FHb <500 mg/l); Grade 1, light hemolysis (500 mg/l ≤FHb <1000 mg/l); Grade 2, medium hemolysis (1000 mg/l ≤FHb <2000 mg/l); and Grade 3, serious hemolysis. If the condition of any sample reached or went beyond Grade 2, the sample was not included in the experiment.

### Erythrocyte morphology observation

An S-4500N scanning electron microscope (Hitachi, Tokyo, Japan) was used to examine the morphology of erythrocytes in the control sample.

### Survey of bacterial pollution in IQC products

The supernatant from each tube of IQC product was observed for any colony. Special agglutination and obvious color changes were observed.

### Statistical analysis

The data obtained were presented as the mean ± standard deviation (SD). A t-test was used to compare the time data of the two groups. One-way analysis of variance (ANOVA) was used to compare the data on various preservation periods within the same sample. P<0.05 was considered to indicate a statistically significant result.

## Results

### Reaction activity of the antigen

**Figure 1:**
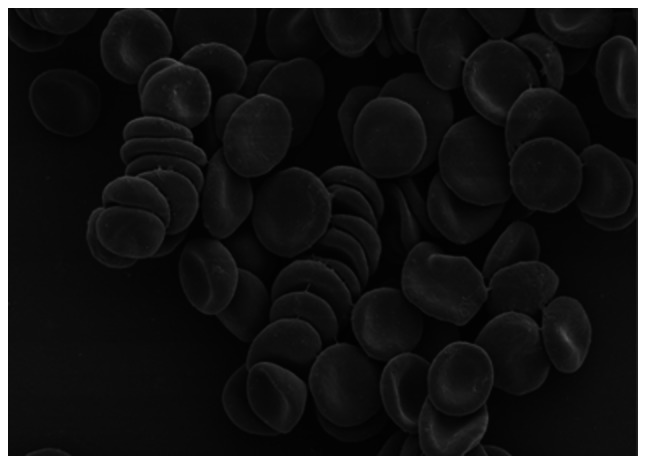
Morphology of erythrocytes before washing and preservation with a scanning electron microscope.

During preservation, no obvious change (P>0.05) in agglutination was observed between the hematocrit of the RBC B antigen and standard anti-B serum in the two groups. In addition, no apparent difference (P>0.05) was observed between the two groups within the same preservation period. Fluctuation (P<0.01) occurred in the hematocrit of the agglutination of the IgM anti-A antibody and reverse grouping RBC. Likewise, no obvious difference (P>0.05) was found between the two groups preserved for an equal length of time. No change (P>0.05) was observed in the agglutination of IgG anti-D antibody and O-group RhD-positive RBC. Finally, no obvious difference (P>0.05) was found in the two groups preserved for the same length of time (Fig. 1).

### Changes in biochemical indicators

During the preserving process, the Na^+^ concentration of the supernatant of both groups continuously declined as the preservation period lengthened. Both groups were preserved for 0 days and the saline group was found to have a higher Na^+^ concentration than the PS sample (P<0.01). No difference was observed between the two samples when preserved for 35 or 42 days. A longer preservation period resulted in higher supernatant K^+^ in the samples (P<0.01). Meanwhile, no obvious difference (P>0.05) was observed between the two groups preserved for 0, 35 or 42 days. The supernatant LDH concentration of the two groups increased slightly (P>0.05). The lactic acid concentration in the supernatant of the two groups increased (P<0.01). The FHb content in the supernatant of two groups increased as the preservation period lengthened. When preserved for 49 days, the increment reached its peak (P<0.01). The FHb in some IQC products reached Grade 2 hemolysis, with no obvious difference (P>0.05) between the two groups preserved for 0, 35, 42 and 49 days (Table I).

### Changes in erythrocyte morphology

**Figure 2:**
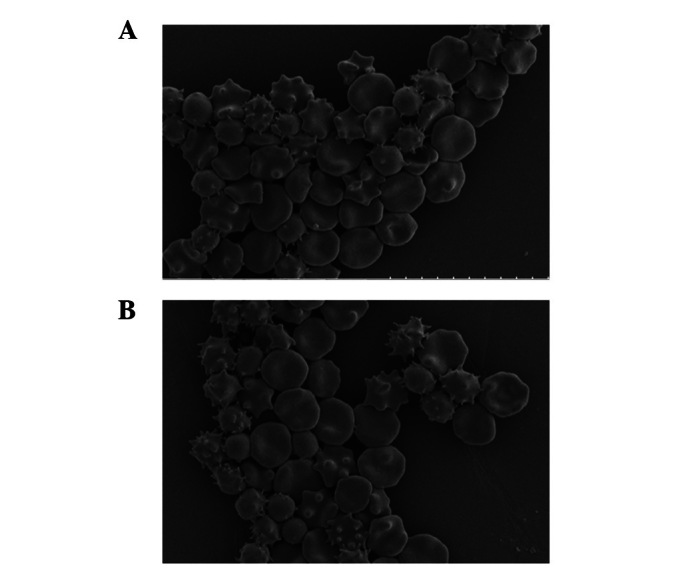
Morphology of erythrocytes preserved for 35 days in the preservative (PS) group (A) and the saline group (B) with a scanning electron microscope.

**Figure 3:**
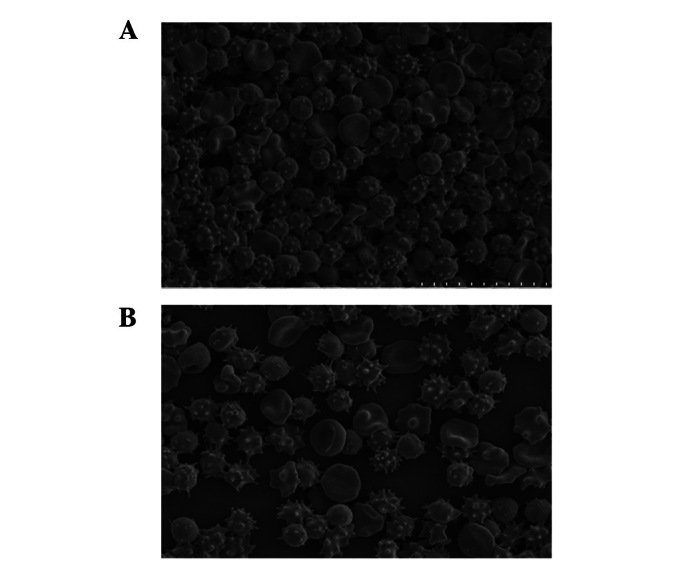
Morphology of erythrocytes preserved for 42 days in the preservative (PS) group (A) and the saline group (B) with a scanning electron microscope.

The morphology of most erythrocytes in the mixed control resembled a double-cave round-shaped disk (Fig. 1). When the control was observed after 35 days, significant crimple and spinous processes were found on ∼30–40% of the erythrocytes of the PS and saline groups (Fig. 2). Moreover, no obvious difference was found between the two groups. After 42 days, significant crimples and spinous processes were found on ∼70–80% of erythrocytes of the PS and saline groups (Fig. 3). Similarly, no distinct difference was observed between the two groups.

### Bacterial pollution observation on IQC products

No bacteria colony, obvious RBC agglutination or color change was observed.

## Discussion

IQC is an important measure in ensuring precise medical laboratory test results and is also a basic requirement (12) of the Accreditation Criteria for the Quality and Competence of Medical Laboratories. The use of approved IQC products is one of the key factors to having guaranteed QC results. Ideally, IQC products should be kept in the same preservation condition as the examined samples to prevent any discrepancy between IQC products and ordinary samples (5,12). The current blood transfusion compatibility testing, especially automated testing, examines the reaction activity of both RBC blood group antigens and serum blood group antibodies. Thus, the test requires IQC products with quality that is consistent with or close to that of ordinary samples. Such products will be suitable for preservation for an extended time and are likely to have low variability.

The IQC products prepared and preserved based on the previous research conducted in our laboratory may be kept for more than 35 days (13). This type of control, however, was prepared from samples extracted from different donors, with different batch numbers. These products are thus only suitable for laboratories requiring small-scale control. If a laboratory has a large-scale requirement for IQC products, the frequent replacement of IQC products or the use of products from different sources and/or of different batches would be unavoidable. Hence, the vial-to-vial variation will reduce the comparability of the laboratory test results. Therefore, minimum vial-to-vial variation must be one of the features of IQC products.

The ideal situation would be to have absolutely no vial-to-vial variation. When preparing IQC products for blood transfusion compatibility testing, the operators should make use of the best actual laboratory conditions and the best existing sample resources. Operators should also minimize the difference within the same batch to ensure that the test result will be effective and comparable. Accordingly, blood samples collected from several healthy donors within 10 days of the examination were used. The samples, which can be obtained by almost every blood transfusion compatibility testing laboratory, have no use after their corresponding RBCs have been used on patients. The samples were mixed on the basis of their blood groups and were then washed and centrifuged to obtain the raw material for IQC products, which are relatively consistent in both RBC blood group antigen and serum antibody. Naturally, the raw material is a necessity for IQC product preparation.

The stability of the analytic indicators is the most important feature of IQC products and equally important is the elimination of interference factors (14–16). The research showed that washing the raw material of IQC products is necessary to remove protein agglutination and some aged RBC segments from the RBC-reducing non-specific interference during control. Conversely, the washing process may eliminate the lactic acid accumulated on the RBC for better preservation. Changes in Na^+^, K^+^, LDH, lactic acid and FHb in the supernatant of the control are important indices that reveal RBC damage. Traditionally, saline-washed RBC is believed to be preserved for only 24 h since hemolysis occurs during prolonged preservation. In the present research, saline and isotonic MAP RBC preservative were used to wash the RBC material. The K^+^, LDH and lactic acid contents of both groups increased as the preservation time increased (P<0.01 or P>0.05, Table I). However, no obvious difference was observed between the two groups when preserved for 35 and 42 days (P>0.05, Table I).

Na^+^ content decreased during the same extended period (P<0.01, Table I), which can be attributed to the RBC preservation injury that induced K^+^ to flow out and be exchanged with Na^+^. When preserved for 0 and 42 days, the Na^+^ content in saline samples was higher than that in the PS group (P<0.01, Table I). This finding may be attributed to the inherent difference in Na^+^ content of the samples from the two groups.

The FHb content in both groups increased with prolonged preservation time. The peak occurred at 49 days (P<0.01, Table I) and no obvious statistical difference was found between the two groups (P>0.05, Table I). Moreover, no obvious change in the agglutination of RBC B antigen and anti-B serum, or in the IgG anti-D antibody and group O RhD-positive RBC (P>0.05, Table I) was observed between the two groups after being preserved for an equal period of time (P>0.05, Table I). Although fluctuations occurred in the agglutination of the IgM anti-A antibody and reverse-grouping A cell (P<0.01, Table I), the change of agglutination strength was no more than 1+, which may be due to systematic errors, thus satisfying the basic requirements of IQC.

The result of scanning electron microscopy showed that the number of crimples was increasing and that spinous processes occurred as the preservation prolonged, but no obvious difference was found between the two groups preserved for the same time. Thus, we believe that no obvious difference arises when using saline and isotonic RBC preservative to wash the RBC material.

In the late preservation time of IQC products (∼42 days), injury occurred on RBC to some extent, including an increase in K^+^, LDH, lactic acid and FHb, and a change in RBC shape. However, no obvious impact on the main performance indices of IQC products was observed ,and the reactive behavior of the RBC blood group antigen and serum antibody was maintained.

When IQC products were preserved for 49 days, the remaining supernatant in every sample was insufficient. Thus, only one biochemical index, FHb, was tested. The test showed that certain IQC products reached Grade 2 hemolysis. The above findings prove that whole blood IQC products retained good stability within 42 days.

The addition of an RBC preservative into whole blood or RBC may effectively extend the shelf life of blood products (17–19). To preserve whole blood IQC products that contain RBC effectively, the substance that may provide RBC metabolic energy, which acts against hemolysis, should be added. IQC products are usually kept in an open environment at 4°C.

Bacteriostasis should also be considered. The initial research showed that whole blood IQC products with added glucose, adenine and mannite were preserved at 4°C for over 35 days. In this research, the washing hematocrit RBC and RBC preservative were mixed with plasma at 1:2:3. The mixed liquid was then placed into hard plastic tubes, each of which contained 6 ml control. Then, 0.05 mg/ml gentamicin sulphate was added.

The difference between IQC products prepared using the proposed method and the ordinary test samples was evident in the supernatant (RBC preservative and plasma). The supernatant of the former was more significant than that of the latter. The amount of plasma used for IQC products is significantly greater than that used for hematocrit RBC. This feature of IQC products enables the preservation and increases the utilization efficiency of RBC. Although the addition of preservative into IQC products may dilute the antibody to a certain extent, the effect on the reactive intensity remains within the acceptable range. Moreover, the weakly positive antibody control material caused by dilution contributed to the monitoring of the sensitivity of detection system. Clean containers and a bacteria-free solution were used in the preparation and some antibiotics were added to prevent bacteria from growing. The IQC products in the two groups could be effectively preserved for more than 42 days.

In summary, IQC products prepared using the proposed technique have the advantages of long preservation time, low vial-to-vial variation and stable antigen and antibody reaction activity, thus satisfying the requirements of blood transfusion compatibility testing. Such IQC products are suitable for application in the field of blood transfusion compatibility testing.

## Figures and Tables

**Table I:** Parameter changes of prepared controls in the preservative (PS) and saline groups during preservation (mean ± SD, n=10).

Parameter	Time (days)	PS group	Saline group
B antigen (Score)	0	12.0±0	12.0±0
	35	12.0±0	12.0±0
	42	12.0±0	12.0±0
	49	12.0±0	12.0±0
IgM anti-A antibody (Score)	0	11.0±1.1	11.3±1.0
	35	11.3±1.0	11.7±0.8
	42	12.0±0^c^	11.7±0.8
	49	12.0±0^c^	12.0±0^c^
IgG anti-D antibody (Score)	0	8.0±0	8.0±0
	35	8.0±0	8.0±0
	42	7.5±1.2	7.5±1.2
	49	8.0±0	8.0±0
Na^+^ (mmol/l)	0	139.8±1.9	141.8±1.0^b^
	35	129.8±2.6^c^	131.6±4.1^c^
	42	124.9±1.9^c^	126.8±1.4^a,c^
	49	-	-
K^+^ (mmol/l)	0	9.2±1.4	9.4±1.1
	35	12.7±1.3^c^	12.5±1.3^c^
	42	14.4±1.4^c^	14.3±1.7^c^
	49	-	-
LDH (U/l)	0	241.1±40.6	247.0±34.6
	35	262.9±43.1	280.4±55.1
	42	278.7±51.6	287.5±58.9
	49	-	-
Lactate (mmol/l)	0	8.4±2.2	8.5±1.7
	35	10.7±1.5^c^	11.3±1.0^c^
	42	10.9±1.6^c^	11.6±1.1^c^
	49	-	-
FHb (mg/l)	0	72.8±7.7	69.1±18.3
	35	94.5±33.5	75.8±32.9
	42	159.4±51.9	182.2±109.9
	49	857.1±301.5^c^	595.3±334.9^c^

a P<0.05,

b P<0.01, vs. preservative (PS) group;

c P<0.01, vs. results of day 0; -, not detected due to insufficient supernatant.
